# Intestinal microbial communities and *Holdemanella* isolated from HIV+/− men who have sex with men increase frequencies of lamina propria CCR5^+^ CD4^+^ T cells

**DOI:** 10.1080/19490976.2021.1997292

**Published:** 2021-11-24

**Authors:** Eiko Yamada, Casey G. Martin, Nancy Moreno-Huizar, Jennifer Fouquier, C. Preston Neff, Sara L. Coleman, Jennifer M. Schneider, Jonathan Huber, Nichole M. Nusbacher, Martin McCarter, Thomas B. Campbell, Catherine A. Lozupone, Brent E. Palmer

**Affiliations:** a Division of Allergy and Clinical Immunology, Department of Medicine, University of Colorado Anschutz, Aurora, Colorado, USA; b Division of Biomedical Informatics and Personalized Medicine, Department of Medicine, University of Colorado Anschutz, Aurora, Colorado, USA; c La Jolla Institute of Immunology, La Jolla, California, USA; d Department of Surgery, University of Colorado Anschutz, Aurora, Colorado, USA; e Division of Infectious Diseases, Department of Medicine, University of Colorado Anschutz, Aurora, Colorado, USA

**Keywords:** Gut, HIV transmission, microbiome, MSM, T-cell recruitment, CCR5

## Abstract

Men who have sex with men (MSM), regardless of HIV infection status, have an intestinal microbiome that is compositionally distinct from men who have sex with women (MSW) and women. We recently showed HIV-negative MSM have elevated levels of intestinal CD4^+^ T cells expressing CCR5, a critical co-receptor for HIV. Whether elevated expression of CCR5 is driven by the altered gut microbiome composition in MSM has not been explored. Here we used *in vitro* stimulation of gut Lamina Propria Mononuclear Cells (LPMCs) with whole intact microbial cells isolated from stool to demonstrate that fecal bacterial communities (FBCs) from HIV-positive/negative MSM induced higher frequencies of CCR5^+^ CD4^+^ T cells compared to FBCs from HIV-negative MSW and women. To identify potential microbial drivers, we related the frequency of CCR5^+^ CD4^+^ T cells to the abundance of individual microbial taxa in rectal biopsy of HIV-positive/negative MSM and controls, and *Holdemanella biformis* was strongly associated with increased frequency of CCR5^+^ CD4^+^ T cells. We used *in vitro* stimulation of gut LPMCs with the type strain of *H. biformis*, a second strain of *H.* *biformis* and an isolate of the closely related Holdemanella porci , cultured from either a HIV-positive or a HIV-negative MSM stool. *H. porci* elevated the frequency of both CCR5^+^ CD4^+^ T cells and the ratio of TNF-α/IL-10 Genomic comparisons of the 3 *Holdemanella* isolates revealed unique cell wall and capsular components, which may be responsible for their differences in immunogenicity. These findings describe a novel mechanism potentially linking intestinal dysbiosis in MSM to HIV transmission and mucosal pathogenesis.

## Introduction

Studies of feces and mucosal biopsies have revealed that high-risk men who have sex with men (MSM) have pronounced differences in gut microbiome composition compared with women and men who have sex with women (MSW), even in the absence of HIV infection.^1–5^ These differences have most notably been characterized by an enrichment of the bacterial genus *Prevotella* and a depletion of the genus *Bacteroides* in MSM, but there are many other differentiating taxa.^4^ Microbiome differences in MSM are interesting in light of published findings that have shown HIV-negative MSM also exhibit immune differences, such as higher blood T cell activation,^5^ increased endotoxemia,^6^ and increased T cell pro-inflammatory cytokine production in colon mucosa.^3^ Our group has reported that the MSM-associated microbiome directly stimulates activation of immune cells *in vitro*^1^ and in gnotobiotic mice.^2^ Furthermore, exposure of Lamina Propria Mononuclear Cells (LPMC) from resected gut tissue to whole intact bacteria isolated from fecal slurries by density gradient centrifugation (fecal bacteria communities; FBCs) of MSM increased *in vitro* HIV infection compared to LPMCs exposed to bacteria from MSW and women. These results implicate the MSM-associated gut microbiome as a potential risk factor for transmission of HIV via anal intercourse, but the mechanism of how the MSM-associated microbiome impact levels of HIV infection and particular microbes enriched in the MSM-microbiome that could elevate HIV infection, remain unclear.

CD4^+^ T cells that express the HIV co-receptor, C-C chemokine receptor type 5 (CCR5), are an important target of HIV.^7–9^ CCR5^+^ CD4^+^ T cells are very abundant in gut mucosal tissue, an important site of transmission via anal intercourse in MSM,^10^ and these cells potentially traffic to both lymphoid and nonlymphoid tissues in many parts of the body, consistent with the widespread anatomical extent of HIV-1 infection.^11^ Also, it has been reported that activated CCR5^+^ CD4^+^ T cells are elevated in gut associated lymphoid tissue (GALT) both during primary HIV infection^12,13^ and once HIV infection is established.^14^ We have previously shown that MSM have a greater number of CD4^+^ T cells expressing CCR5 in rectal biopsy samples compared with MSW, which may be a risk factor for HIV transmission in MSM.^15^ However, it is not clear whether differences in microbiome composition drive elevated levels of these cells and if so, which particular microbes, expressed genes, or host responses are involved.

In this study, we investigated whether FBCs of MSM were a causal factor in the observed increased frequency of CCR5^+^ CD4^+^ T cells using *in vitro* stimulation of gut LPMCs. To further understand which individual microbial taxa may be important, we identifiedtaxa (characterized with 16S rRNA targeted sequencing) that correlated with the frequency of CCR5^+^ CD4^+^ T cells in rectal biopsy samples from HIV-positive/negative MSM. We next validated the ability of different isolates of one of the identified taxa, *Holdemanella*, to induce CCR5 expression on CD4^+^ T cells using *in vitro* stimulation of LPMCs and related stimulatory activity to genome content to predict potential mechanisms of variation. This study provides evidence that increased frequencies of CCR5^+^ CD4^+^ T cells may be due in part to exposure to *Holdemanella* species in the gut of MSM.

## Results

### FBCs from MSM with and without HIV infection increase CCR5^+^ CD4^+^ T cell frequencies

**Figure 1.**
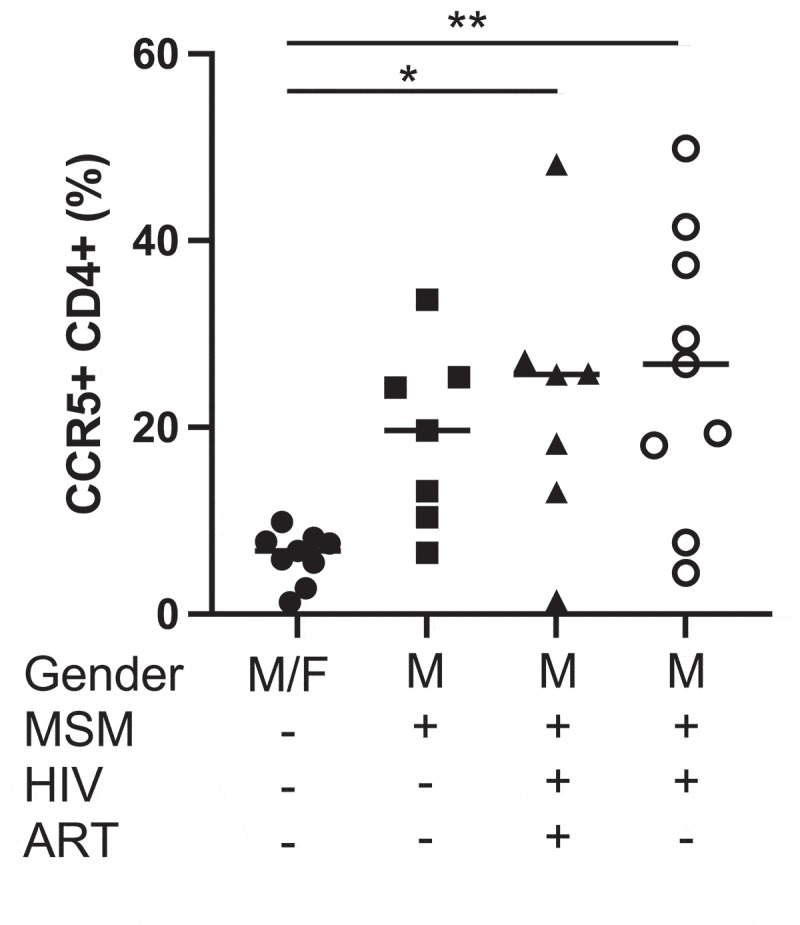
Frequency of lamina propria CCR5^+^ CD4^+^ T cells cultured with FBCs. LPMC cultured with HIV-positive ART−/+ MSM FBC showed significantly higher frequencies of CCR5^+^ CD4^+^ T cells compared to LPMC with HIV-negative non-MSM FBC using Kruskal-Wallis test and Dunn’s multiple comparisons test. * = *p* < .05, ** = *p* < .01

In a prior study we had established that rectal biopsy samples from HIV-positive and negative MSM exhibited higher levels of CCR5^+^ CD4^+^ T cells compared to those from non-MSM.^15^ To establish whether the gut microbiome is a driver of these differences, we performed *in vitro* stimulations of LPMCs with FBCs and compared levels of CCR5^+^ CD4^+^ T cells across groups. To generate FBCs, whole intact bacteria were isolated from fecal slurries by density gradient centrifugation as described by our group previously.^1^ A total of 32 FBCs were collected from HIV-negative non-MSM (n = 9) and MSM (n = 7), HIV-positive individuals on antiretroviral therapy (ART) (n = 7) and off ART (n = 9). Demographics for our study population are shown in Table S1. The gating strategy for viable CCR5^+^ CD4^+^ T cells is shown in Figure S1. We found that the frequency of CCR5^+^ CD4^+^ T cells were higher in LPMCs stimulated by FBC from HIV-positive on and off ART MSM compared to non-MSM (*p* = .028, *p* = .005, respectively). There was also a trend of higher CCR5 upregulation by FBC from HIV-negative MSM compared to HIV-negative non-MSM (Figure 1).

### Diverse bacteria correlate with CCR5^+^ CD4^+^ T cell frequencies in human gut biopsy samples

**Figure 2.**
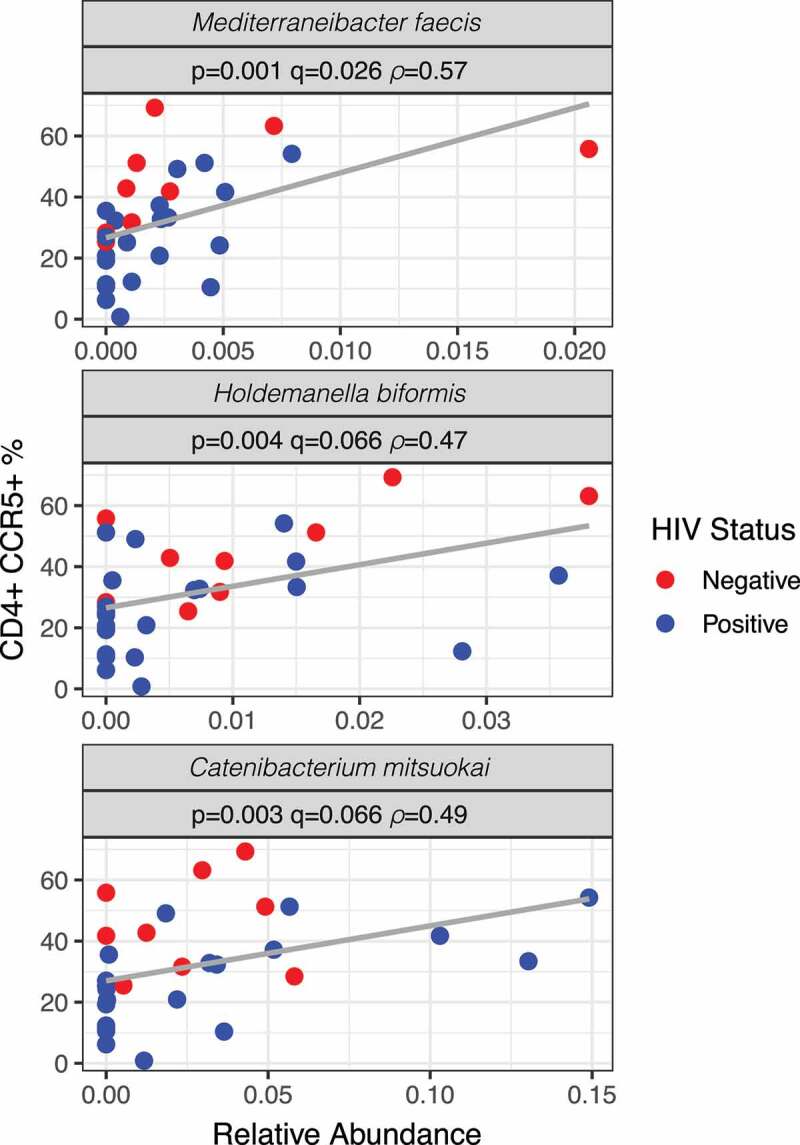
Relationship between the frequencies of colonic CCR5^+^ CD4^+^ T cells and specific bacterial species. Spearman rank correlation was performed using all taxa found in at least half of the samples and with a total frequency of at least 10. Amplicon sequence variants (ASVs) were first identified using DADA2 and clustered at 97% similarity using VSEARCH with closed reference OTU picking using the Greengenes database. Scatterplots are shown for the three taxa with significance after correction for multiple comparisons (q < 0.1). HIV+ individuals are in blue and HIV− individuals are in red. Note that the “y” axis is evenly scaled but each “x” axis is different for each taxon

To determine whether the frequency of CCR5^+^ CD4^+^ T cells correlated with specific intestinal bacteria, we performed both Cytometry by Time-Of-Flight (CyTOF) and 16S rRNA targeted sequencing on a total of 30 rectal biopsy samples from HIV-negative MSM (n = 9), and HIV-positive MSM on ART (n = 21). Operational Taxonomic Units (OTUs picked at 97% threshold after denoising with DADA2 (see methods)) were defined and those that were identified in at least half of the samples and with a total frequency of at least ten were used for Spearman rank correlation with correction for multiple comparisons with FDR.^16^ Three different OTUs positively correlated with CCR5^+^ CD4^+^ T cells (q < 0.1; Figure 2). These included *Mediterraneibacter faecis* (*p* = .001), *Holdemanella biformis* (formally *Eubacterium biforme;*^17^ *p* = .004) and *Catenibacterium mitsuokai* (*p* = .003), the last two of which have been shown to be enriched in HIV-negative MSM compared to MSW.^4^

### *H.* *porci* elevates the frequency of CCR5^+^ CD4^+^ T cells and elicits cytokine production with a high TNF-α/IL-10 ratio

While three OTUs were found to be associated with elevated frequencies of CCR5^+^ CD4^+^ T cells and enriched in the MSM microbiome, we chose to focus further experiments on *H. biformis* and closely related *H. porci* because *H. biformis* has been frequently found to correlate with immune cell activation in our prior studies. Specifically, in an analysis of the blood of HIV-positive and negative MSM, an Amplicon Sequence Variant (ASV) with a 99.3% identity to type strain (ATCC strain VPI C17-5) of *H. biformis*, positively correlated with CD8^+^ T cell activation (HLADR^+^ CD38^+^ CD8^+^ and CD103^+^ CD8^+^) and CD4^+^ T cell homing (CD103^+^ CD4^+^).^2^ Furthermore, in gnotobiotic mice gavaged with HIV-positive and negative MSM stools, an ASV with 98% identity to type strain *H. biformis* positively correlated with levels of CD69^+^ CD8^+^ T cells and CD103^+^ CD8^+^ and CD103^+^ CD4^+^ T cells (indicating increased immune activation and gut homing) in the mice. We had also shown previously that the type strain of *H. biformis* (VPI C17-5; ATCC 27806) induced an inflammatory cytokine profile, characterized by higher tumor necrosis factor α (TNF-α)/interleukin 10 (IL-10) ratio, when used to stimulate Peripheral Blood Mononuclear Cells (PBMCs) of HIV-positive and negative individuals compared to LPS or several other gut commensal bacteria tested, including *Bacteroides fluxus, Bacteroides dorei*, and *Prevotella copri*.^18^ Finally, our ongoing efforts to culture a diversity of different intestinal bacteria out of HIV-positive and negative MSM stools had resulted in the isolation into pure culture of *H. biformis* (97% ID to type strain) and closely related *H. porci* (95% ID to *H. biformis* type strain), providing the opportunity to investigate the activity and genomic content of strains relevant to MSM. One of these novel strains is from an HIV-negative MSM stool (*H. porci*) and one from a HIV-positive ART+ MSM stool (*H. biformis* strain 2).

**Figure 3.**
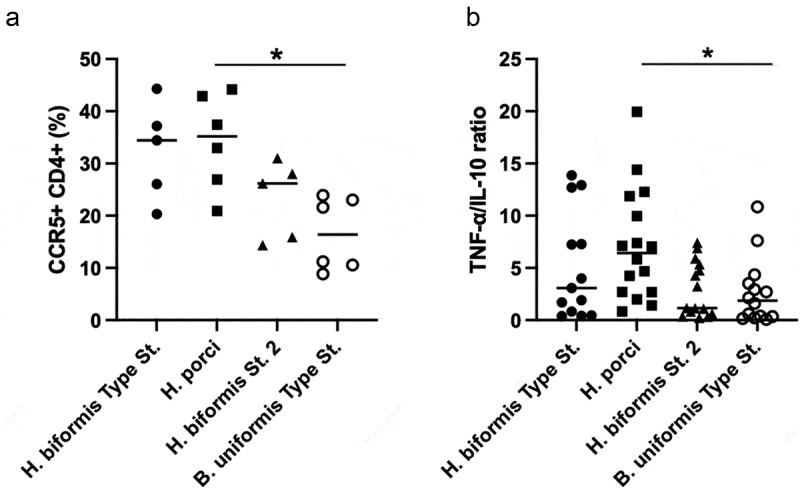
*H. porci* elevated the frequency of CCR5^+^ CD4^+^ T cells and TNF-α /IL-10 ratio in LPMC. A) LPMC cultured with *H. porci* showed significantly higher frequencies of CCR5^+^ CD4^+^ T cells expressing the HIV co-receptor CCR5 compared to LPMC with *B. uniformis*. B) TNF-α to IL-10 ratio after 24 hours stimulation showed *H. porci* upregulated a significantly higher TNF-α/IL-10 ratio compared to *B. uniformis*. Statistical significance was determined using the Kruskal-Wallis test and Dunn’s multiple comparisons test. * = *p* < .05

To test the immune-stimulatory activity of *Holdemanella isolates*, we stimulated LPMCs from resected gut tissue with heat killed bacteria, including the type strain *H. biformis* (VPI C17-5; ATCC 27806) and our two novel *Holdemanella* isolates. For comparison, we also stimulated LPMCs with the type strain *Bacteroides uniformis* and a cell culture media control. *B. uniformis* was chosen because we had previously found that this microbe was decreased in HIV-positive and negative MSM,^1,2,4^ and negatively correlated with CD4^+^ T cell levels in the ileum of gnotobiotic mice colonized with MSM stools and with CD4^+^ T cells expressing the gut homing marker CD103.^2^ Viable CCR5^+^ CD4^+^ T cells were identified using the gating strategy shown in Figure S1. While CCR5 expression was elevated in LPMCs cultured with all *Holdemanella* isolates, *H. porci* was the only one that was significantly higher when compared to *B. uniformis* (*p* = .015, Figure 3a). *H. biformis* type strain was the next highest, while strain 2 induced the lowest CCR5 expression compared to the other two *Holdemanella* isolates. Because we have previously shown that *H. biformis* type strain induced a significantly higher ratio of pro-inflammatory TNF-α to anti-inflammatory IL-10 than all other gut bacteria we have previously examined^18^ and because these cytokines have both been shown to modulate CCR5 expression,^19,20^ we measured the levels of these cytokines in the supernatant. Similar to CCR5 expression, cytokine concentrations in LPMCs cultured with strain 1 exhibited a significantly higher TNF-α/IL-10 ratio when compared with *B. uniformis*, (*p* = .012, Figure 3b) followed by the type strain and then strain 2. Collectively, these data suggest that *Holdemanella* modulates both CCR5 expression and a pro-inflammatory cytokine profile in a similar strain dependent manner, although the association between the CCR5 and TNF-α/IL-10 ratio is relatively weak suggesting other factors are involved.

### Comparative genomics of *Holdemanella* isolates

**Figure 4.**
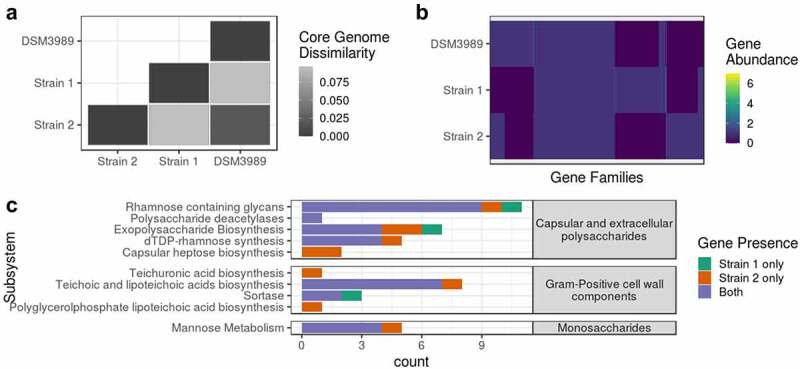
Genomic differences in *Holdemanella* isolates. Genome sequencing of *Holdemanella* isolates revealed heterogeneity. The cophenetic distance matrix for the persistent genome alignment is given in Panel A. Dissimilarity is shown as FastTree’s adjusted estimate of protein similarity as scored by BLOSUM45. Panel B is a heatmap that depicts the variation in abundance of gene families (n = 3753) by strain. Panel C compares the presence of genes involved in cell membrane and wall biosynthesis as well as mannose metabolism in both isolates

**Figure 5.**
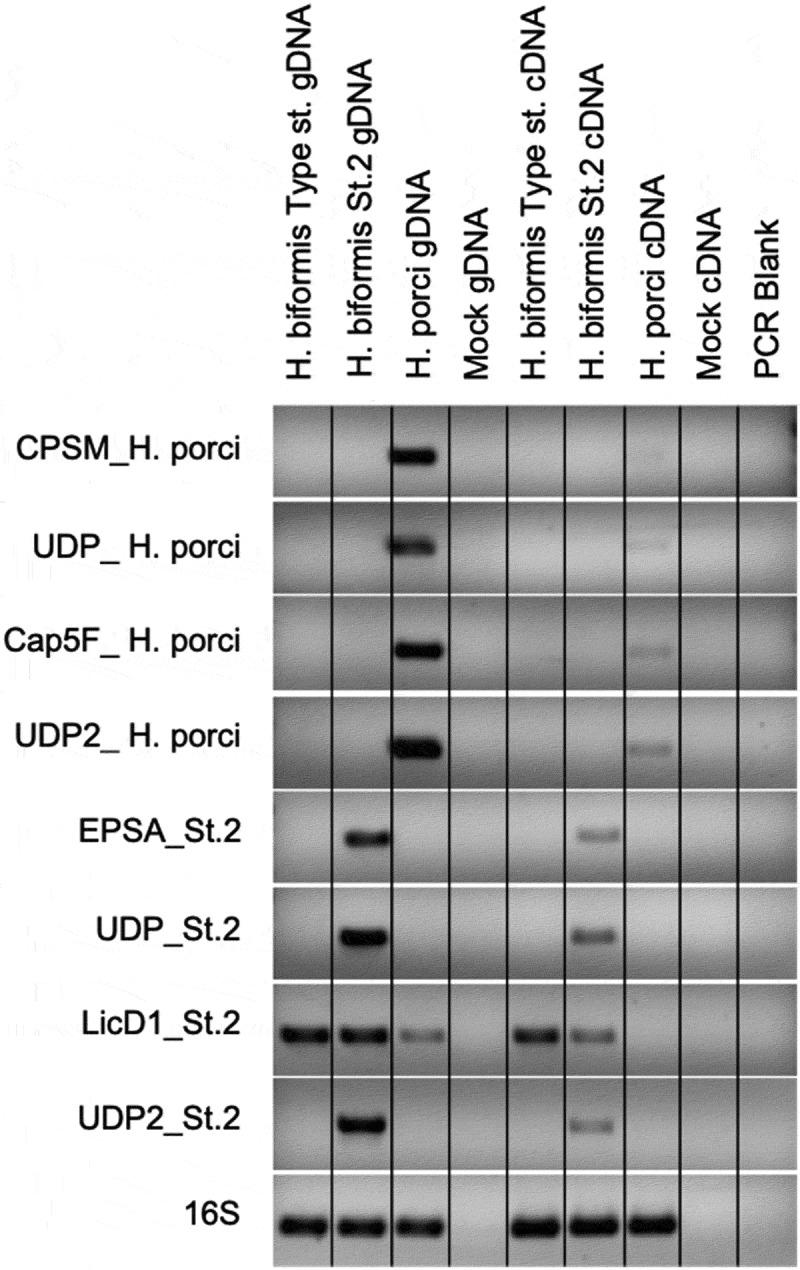
Expression of unique genes of cell surface factors. Agarose gel electrophoresis (1% agarose) of amplified PCR product using gene specific primers and 16S V4 (515 F:806 R) primers for positive control. Genomic DNA and cDNA templates were used. Positive bands for 7 of 8 primer pairs show both presence of gene in gDNA and gene expression in cDNA for the corresponding strain putatively determined from in silico analysis. licD1, thought to only be present in strain 2, was detected in the gDNA in all 3 strains while gene expression was observed in Type strain and strain 2. Gene expression for the four strain 1 specific genes was low, but faint bands can be seen. Primer sequences and predicted function gene function can be found in Table S2

Very little is known about the biology of *Holdemanella*, which is a member of the poorly characterized Erysipelotrichaceae family of the Clostridiales. *H. biformis* was first described as a part of the normal gut flora in 1974.^21^ A draft genome had been generated for the type strain of *H. biformis* and deposited in NCBI (PRJNA29269), but not analyzed deeply. To investigate potential mechanisms underlying variation across *Holdemanella* isolates in the stimulation of CD4^+^ CCR5^+^ T cells and in TNF-α/IL-10 ratio, we additionally sequenced the genomes of the two novel isolates. The genomes of the *H. biformis* type strain (DSM3989),*H. biformis* strain 2 and *H. porci* had an estimated completeness of 96.8%, 98.1%, and 98.1% respectively. We identified a core genome of 1410 genes (~60% of the gene family content of each isolate'sgenome) across the 3 bacteria. Alignment of the core genome’s translated products revealed that *H. porci* was the most distantly related of the three isolates, with the *H. biformis* type strain and strain 2 being more closely related (Figure 4a). The pangenome spanned 3752 gene families, of which, ~50% (1836 genes) were isolate-specific and ~13% (485 genes) were common to two isolates. These differences in coding capacity at the gene family are shown in Figure 4b. Interestingly, unique gene memberships were concentrated in pathways involved in forming capsular polysaccharides and components of the gram-positive cell wall (Figure 4c). Because these are draft and not complete genomes, and these differentially present genes might not be expressed in the growth conditions that we employed, we used PCR and RT-PCR to confirm differential presence and expression of a subset of the genes that differentiated *H. porci* from *H. biformis* strain 2. Specifically, for each bacteria, we identified four unique genes involved in the synthesis and regulation of cell surface factors (testing 8 genes total; Figure 5). Details about the predicted functions for these genes are in Table S2. Expression of 16S rRNA was used as a positive control. Of the 8 genes tested, 7 were only found in the expected isolate, and were additionally not found in the type strain. The one exception (LicD1_S2) had a lighter PCR band in *H. porci*. This could either be because of nonspecific amplification or because the gene was in a gap region of this draft genome. The RT-PCR results indicated that 6 of the 8 genes were clearly expressed, while two produced only very faint bands. Taken together, these results suggest that a number of surface factor genes that we predicted to differentiate *H. porci* from *H. biformis* strain 2 are truly differentially present and expressed.

### Role of cytokines in increasing CCR5^+^ CD4^+^ T cell frequency

**Figure 6.**
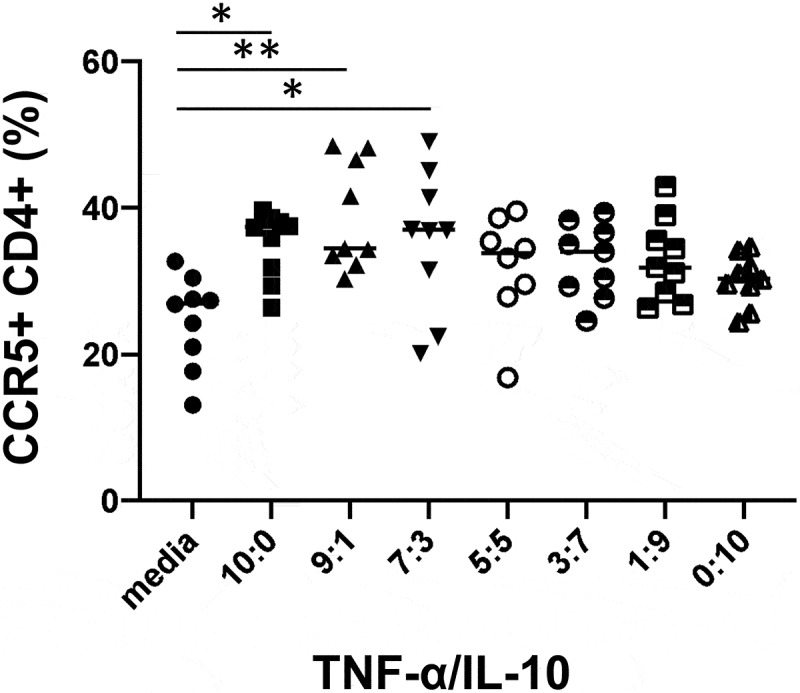
Characterization of immune responses to TNF-α/IL-10 ratio in LPMC.Frequencies of CCR5^+^ CD4^+^ T cells were significantly upregulated by higher TNF-α/IL-10 ratio stimulation compared to media controls, 10:1, 9:1 and 7:3 TNF-α:IL-10 (*p* = .0109, *p* = .0011, *p* = .0123, respectively). Statistical significance was determined using Kruskal-Wallis test and Dunn’s multiple comparisons test. * *p* < .05, ** *p* < .01

We had previously found that stimulation of PBMC with the type strain of *H. biformis* resulted in a significantly higher TNF-α/IL-10 ratio in culture supernatant compared to several other bacteria tested.^18^ Furthermore, stimulations of LPMC performed here exhibited significantly higher TNF-α/IL-10 by *H. porci* (Figure 3b). Because it had been previously shown that both TNF-α and IL-10 can regulate CCR5 expression,^19,20^ and stimulation by only TNF-α didn’t upregulate in this study (data not shown), we wanted to determine whether a high ratio of TNF-α to IL-10 would impact the frequency of CCR5^+^ CD4^+^ T cells. To explore this question, we used exogenously added TNF-α and IL-10 to stimulate LPMCs using several different ratios. Cultures were incubated with both cytokines across a gradient because it has been shown that TNF-α can induce CCR5, while IL-10 inhibits its expression, thus examining the effects of both concurrently is biologically relevant as *Holdemanella* induces both in cultures. Significant increase in CCR5^+^ CD4^+^ T cells was seen in 10:0, 9:1 and 7:3 TNF-α/IL-10 ratios (*p* = .011, *p* = .001, *p* = .012, respectively, Figure 6) compared to media alone. However, there was no statistically significant change in CCR5 expression when only IL-10 was added (0:10) or ratios of cytokine which contain higher amounts of IL-10 (1:9, 3:7 or 5:5) so it appears that TNF-α is driving the increase and IL-10 is not inhibiting CCR5 expression (Figure 6). This experiment was repeated twice using the same LPMC sample.

## Discussion

We had previously reported differences in the gut microbiome in HIV-positive and high-risk MSM, and used fecal microbiota transplantation into gnotobiotic mice, *in vitro* stimulation assays and HIV infection assays with FBCs to support a direct role for the MSM-associated gut microbiota in immune activation and increased HIV infection of CD4^+^ T cells.^1,2^ We had also previously shown that HIV-positive and high-risk MSM had elevated frequencies of the HIV co-receptor CCR5^+^ on CD4^+^ T cells in human gut biopsies.^15^ Since MSM-associated factors such as trauma, douche, PrEP and antibiotics can also enhance HIV infection and CCR5 expression on CD4 + T cells in MSM, here we performed *in vitro* experiments specifically designed to examine the effect of the microbiome alone.

We found a direct role of MSM-associated gut microbiomes overall and of *Holdemanella* specifically in driving CCR5 expression on CD4^+^ T cells, indicating that an MSM-microbiome induced increase in HIV co-receptor expression on CD4^+^ T cells may be one factor driving increased infection. We also provide data potentially linking the TNF-α/IL-10 cytokine ratio to this process. The concept that an altered microbiome can influence rectal HIV transmission via induction of CCR5 expression on CD4^+^ T cells is supported by two recent studies in macaques.^22,23^ Specifically, rhesus macaques from two different sources had significantly different rates of infection against repeated low-dose SHIV intrarectal challenge, and this was associated with an altered gut microbiome and an increase in activated CD4^+^ CCR5^+^ Ki67^+^ T cells in the rectal mucosa of the susceptible group.^22^ A more recent study also supported a link between the gut microbiome and gut CCR5 expression by showing that probiotic intake reduced CCR5 expression on CD4^+^ T cells in the gut of macaques.^23^ These studies together support that MSM-microbiome mediated increases in CD4^+^ CCR5^+^ T cells in the rectum has potential implications for increased risk of HIV transmission and that the gut microbiome may be a viable target for disease prevention. More work will be needed, however, to validate that the gut microbiome of MSM actually induces increased transmission of HIV *in vivo* and the particular microbes and mechanisms involved.

In this study, we demonstrated that *H. biformis* associated with CCR5 expression in rectal biopsies of MSM and that the highly related *H. porci* induced CCR5 expression on intestinal CD4^+^ T cells *in vitro* in a manner that may be related to unique capsular and cell wall components expressed by this organism. This work adds to our prior work showing that *Holdemanella* was increased in HIV-positive and negative high-risk MSM,^1,18^ correlated with inflammatory markers in human blood and gnotobiotic mice,^2^ and induced significantly higher TNF-α/IL-10 ratio when cultured with PBMCs from both HIV-negative and HIV-positive subjects, compared to other gut commensal bacteria including *P. copri* and multiple species in the Bacteroides genus.^18^

Very little is known about the biological attributes of *H. biformis* and its close relatives, which are in the poorly understood Erysipelotrichaceae family of the Clostridiales order. *H. porci* is highly related to *H. biformis* and this species was only recently defined.^24^ *H. biformis* was enriched in individuals with Irritable Bowel Syndrome,^25^ in the active disease stage compared to remission in an individual with Ulcerative Colitis,^26^ with an unhealthy fasting lipid profile in postmenopausal women with obesity,^27^ and with the progression of chronic kidney disease and dialysis.^28^ These studies support that the pro-inflammatory profile that we have described for *H. biformis* in this and previous works^18^ may have broader implications for driving inflammation in diverse disease contexts. However, another study found that *H. biformis* was depleted in advanced human colon adenoma samples and that spent media from *H. biformis* cultures could inhibit tumor formation in mice.^29^ Consistent with beneficial effects being promoted by spent media as opposed to the cells themselves, other studies have found that *H. biformis* can produce anti-inflammatory metabolites including butyrate^30^ and the long chain fatty acid C18-3OH.^31^ Although these findings may appear contradictory, it is not uncommon for pathogens and pathobionts to express both pro- and anti-inflammatory factors to manipulate the immune system.^32–34^

Our comparative analysis of genomes and immune modulatory activity of 3 different isolates of *Holdemanella* suggest that variation in components of the cell envelope and capsule may be responsible for substantial variation in immune modulatory potential. *H. porci* cultured from an HIV-negative MSM induced higher CCR5 expression compared to *H. biformis* strain 2 (cultured from an HIV-positive MSM) and higher TNF-α/IL-10 ratio compared to both the type strain and strain 2 of *H. biformis*. *H. porci* expressed several unique urine diphosphate-conjugated polysaccharide epimerases, suggesting specific capsular or exopolysaccharides biosynthesis potential that could be linked with the increased immunogenicity observed. It could also be that strain 2 has more anti-inflammatory moieties which are absent from *H. porci*. In *H. biformis* strain 2, we observed the expression of *epsA*, a putative transcription factor that regulates the composition of capsular polysaccharides. In *Lactobacillus johnsonii, epsA* was shown to induce the expression of both homopolymeric and heteropolymeric capsular exopolysaccharides.^35^ We detected a preponderance of genes involved in the synthesis of rhamnose-containing glycans in both of our laboratory isolates (Figure 4c). Prior work conducted in *Enterococcus faecalis* and *Streptococcus pyogenes*, which also synthesize rhamnose-containing cell wall polysaccharides, has indicated that even subtle changes in structure can significantly impact virulence.^36,37^ Although we have demonstrated variation in both the induction of immune phenotypes *in* *vitro* and gene content across *Holdemanella* isolates, it is important to consider that these assays and culture conditions do not represent the complex environment of the intestine. Intestinal bacteria will vary their gene expression in a context dependent manner, and immune modulation by capsular factors can differ based on other factors present in the environment. *In vivo* studies, such as longitudinal prospective cohorts that evaluate dynamics over time within individuals, would further validate the significance of these *in vitro* observations. Further work conducted with isolated capsular components or genetically manipulated strains would also need to be done to evaluate the effects of different components of the capsule and cell wall of *Holdemanella* isolates on inflammatory immune phenotypes.

Our analysis of bacteria that correlated with the frequency of CCR5^+^ CD4^+^ T cells in rectal biopsy also identified another positively correlated bacteria, *C. mitsuoki*, that we had previously found to be enriched in MSM compared to MSW.^4^ Like *H. biformis, C. mitsuoki* was also found to positively correlate with CD4^+^ T cell activation and/or gut homing in humans and in gnotobiotic mice.^2^ *C. mitsuoki* has been linked with gut microbiome dysbiosis caused by a high fat and high sugar diet^38^ and an unhealthy fasting lipid profile^27^ in the same study that identified an increase in *H. biformis* in this condition indicating that these bacteria, both in the Erysipelotrichaceae family, may co-distribute in similar disease contexts.

In addition to the three isolatesof *Holdemanella*, we also tested *B. uniformis* because it is one of the species that we previously described to be highly depleted in HIV-negative MSM compared to MSW,^4^ and to correlate negatively with activated, gut homing (CD103^+^) CD4^+^ T cells in HIV-positive and negative MSM and in gnotobiotic mice gavaged with their feces.^2^ Interestingly, *B. uniformis*, a Treg and IL-10 inducer^39^ did not induce CCR5 expression on CD4^+^ T cells in our assays and induced significantly lower TNF-α/IL-10 ratio. *B. uniformis* has been found to be reduced in patients with Crohn’s disease, suggesting an inverse relationship with inflammation.^40^ Furthermore, we have previously found that it has the genes to produce a zwitterionic capsular polysaccharide predicted to have similar anti-inflammatory properties to Polysaccharide A (PSA) of *Bacteroides fragilis*^41^ and induced higher levels of IL-10 and T regulatory cells in *in vitro* stimulations with PBMC compared to intestinal Bacteroides without this factor.^41^

In this work we attempted to broadly link the frequency of CCR5^+^ CD4^+^ T cells with cytokine expression. We showed that the frequency of CCR5^+^ CD4^+^ T cells was associated with the ratio of TNF-α/IL-10, consistent with reports from previous studies conducted in PBMC lymphocytes.^19,20^ CCR5 was originally characterized as a receptor for β-chemokines: macrophage inflammatory protein (MIP)-1α, MIP-1β and RANTES.^42^ These β-chemokines have been shown to be increased by TNF-α, whereas reduced by IL-10,^43–47^ suggesting that TNF-α and IL-10 together regulate CCR5 expression. Also, we had previously demonstrated that higher TNF-α/IL-10 ratio in PBMC after *H. biformis* stimulation compared to several other bacteria tested,^18^ and markedly reduced IL-10 production in PBMCs induced with both *H. biformis* and *C. mitsuoki* compared to a related species in the Erysipelotrichaceae family, *Clostridium spiroforme*.^39^ However, in our assay IL-10 alone, or TNF-α/IL-10 ratios in which IL-10 was the predominant cytokine, there were no significant differences in CCR5 expression, leading us to conclude that TNF-α was the main driver of increased CCR5 expression. In addition, increased CCR5 expression in the presence of TNF-α, while statistically significant, was modest, suggesting other immune factors may be involved.

Taken together, HIV infection in MSM through anal intercourse may be enhanced by MSM-associated bacteria through increased expression of CCR5^+^ on lamina propria CD4^+^ T cells with concomitant T cell activation, demonstrating a novel mechanism linking intestinal dysbiosis to HIV-1 mucosal pathogenesis.

*H. biformis* and *C. mitsuoki* were also reported as bacterial species that increased after long-term pre-exposure prophylaxis (PrEP), suggesting that this might be one driving factor of higher levels that have been observed in MSM. However, we have also found *H. biformis* to be enriched in MSM in our earlier studies where the majority of individuals were not on PrEP^1^ and both *H. biformis* and *C. mitsuoki* were enriched in HIV-positive MSM who were untreated or on a diversity of different ART drugs, indicating enrichment in MSM independent of PrEP. Since PrEP has been linked with an increase in high-risk sexual activities, for instance being associated with higher transmission of other STDs,^48,49^ it is still possible that observed increases in the PrEP treated population could be linked with sexual activity. Taken together, *Holdemanella* is associated with MSM behavior, and our data support that its pro-inflammatory profile may be linked with immune activation and the expression of HIV co-receptors that has the potential to impact HIV transmission and inflammatory comorbidities in this population.

## Conclusions

We observed that the gut microbiota of HIV-negative MSM elevates CCR5 expression on lamina propria CD4^+^ T cells *in vitro*. Furthermore, we identified three species of bacteria, two of which are enriched in MSM compared to MSW, that were all associated with increased frequencies of CCR5^+^ CD4^+^ T cells in rectal biopsies and we demonstrate that a close relative of one of these species, *H. porci*, elevated levels of CCR5^+^ CD4^+^ T cells in a strain dependent manner when used to stimulate healthy LPMC, likely driven by particularcell envelope and capsule components. This work defines a mechanism by which the HIV-negative MSM-associated microbiome may influence the risk of HIV transmission through elevated *Holdemanella* abundance and increased chemokine receptor expression on colonic CD4^+^ T cells. These findings describe a novel mechanism potentially linking intestinal dysbiosis in MSM to HIV transmission and mucosal pathogenesis.

## Materials and methods

### Study subjects

Rectal biopsy and stool samples were collected from HIV-negative non-MSM and MSM, and HIV-positive MSM on and off ART. HIV-negative MSM were recruited from a high-risk cohort assembled for a study of a candidate HIV-1 preventative vaccine.^50^ All enrolled individuals live in metropolitan Denver. Individuals were excluded during recruitment if they weighed <110 pounds or had received antibiotics within the prior 30 days. All subjects used in this study were previously characterized for diet and sexual behavior as part of a larger cohort.^4^ Diet or any particular sexual behavior measured previously were not identified as driving factors of the most prominent microbiome differences between MSM and MSW.^4^

### Ethics statement

Written informed consent was obtained from healthy HIV-positive/negative individuals for use of biopsy and stool. The study protocol for sample collection was approved by the Colorado Multiple Institution Review Board (COMIRB# 14–1595, 15–1692, and 17–1512). All subjects were adults. Primary human LPMC were collected from otherwise discarded macroscopically healthy tissues from gut resection surgery, and they were determined to not be human subject research under COMIRB #14–1184.

### Biopsy sample preparation and CyTOF assay

All participants were asked to prepare their bowel for biopsy using a Fleet Saline enema. Following the enema, 30 pinch biopsies were collected from the rectal region of the colon, approximately 3–10 cm from the anal verge. Two of these pinches were preserved for 16S rRNA targeted sequencing as described below and 23 of these pinches were used for CyTOF. The pinches were digested for 1.5 hours with DNase and collagenase and then filtered through a 70 μm nylon filter as previously described.^2^ Biopsy samples were immediately stained for mass cytometry. For live-dead cell distinction, cells were stained with 2.5 μM cisplatin (Fluidigm, South San Francisco, CA). Cells were then resuspended in 70 μl of barium-free FACS buffer (PBS with 0.1% BSA and 2 mM EDTA) and incubated for 30 min at 4°C with a 30 μl mixture of metal-conjugated antibodies (Abs) focused on T cells. Cells were stained with a DNA intercalator (0.125 μM 191Ir and 193Ir; Fluidigm) and acquired using a CyTOF2 mass cytometer (Fluidigm), CyTOF software v.6.0.626 with noise reduction, a lower convolution threshold of 200, event length limits of 10–150 pushes, a σ value of 3, and a flow rate of 0.045 ml/min. These data were analyzed using FlowJo and the frequencies of CD4^+^ CCR5^+^ were used to identify taxa that were positively correlated.

### 16S rRNA gene sequencing and analysis

DNA was extracted from 2 pinch biopsies using the DNeasy PowerSoil Kit (Qiagen). Sequencing of the V4 region of the 16S rRNA gene was performed using 515:806 R primers on an Illumina MiSeq personal sequencer (Illumina, San Diego, CA) according to the Earth Microbiome Project^51^ protocol. For sequence analysis, Quantitative Insights into Microbial Ecology 2 (QIIME 2) (Ver. 2020.11) was used.^52^ DADA2^53^ was used to identify 230-base, single-end, amplicon sequence variants (ASVs). To reduce the number of comparisons made, ASV feature reduction was performed first by clustering ASVs into OTUs at 97% similarity using VSEARCH^54^ closed-reference OTU picking with the Greengenes database (Ver. 13_5).^55^ Additionally, only OTUs identified in at least half of the samples and with a total frequency of at least 10 were used for Spearman rank correlation. A one-sided hypothesis test was used for identification of microbes that positively correlated with CD4^+^ CCR5^+^ cells. Correction for multiple comparisons was made using the Benjamini-Hochberg procedure,^16^ and significant relationships (q < 0.1) were plotted using R programming^56^ with ggplot2.^57^ The three significant OTUs were classified using the RDP online classifier^58^ referencing type strain isolates with at least 1200 bases. We used the top species match with at least 0.80 RDP SeqMatch score. All three query sequences had a BLAST alignment similarity score of 97% or greater with the corresponding reference strain sequence.

### LPMC collection

All patients signed a release to allow the unrestricted use of discarded tissues for research purposes and all protected patient information was de-identified to the laboratory investigators. Tissues were opened longitudinally, and trimmed of excess fat, the mucosal layer and extraneous material, such as stitched portions, damaged tissues or connective tissues. Gut tissue was then washed with PBS supplemented with Streptomycin, Penicillin, and Amphotericin B and incubated at 37°C with PBS containing 1 mM EDTA for 2 cycles of 45 min to remove epithelial cells. The lamina propria layers were cut into 1 g pieces and incubated while shaking with AIMS V media containing antibiotics, 0.5 mg collagenase D and 10 μg DNase I for 3 cycles of 45 min at 37°C. The digested tissues were collected and washed with PBS and resuspended in RPMI1650 containing 10% human serum. Cells from each interval were pooled together, RBC lysed, and cryopreserved and stored in liquid nitrogen.

### Stool sample collection and FBC preparation

Stool samples were collected by the patient, both on a sterile swab and with a sterile scoop within 48 hours of a clinic visit, then immediately stored in a cooler with −20°C freezer packs. After delivery to the lab, the samples were aliquoted and stored at −80°C before use. To separate whole intact microbes from the rest of the fecal material, stool was subjected to density gradient centrifugation as described previously^59^ with some modifications. Specifically, 2 g of feces were homogenized in 40 mL of sterile PBS by aggressive vortex. The homogenized fecal sample was passed through a 100 μm filter. 80% Histodenz (Sigma) was prepared in PBS and was sterilized by autoclave at 121°C for 15 mins. 20 ml of filtered homogenized fecal sample was overlaid on 5 ml of the 80% Histodenz solution in 2 separate tubes and centrifuged at 10,000 g for 40 min at 4°C. The interphase layers corresponding to the microbiota were transferred to a 50 ml tube and were washed, resuspended, overlaid on 5 ml of 80% Histodenz solution and centrifuged again at 10,000 g for 20 min at 4°C. The top layer was discarded and the microbiota layers were extracted to a new tube and washed with 10 ml PBS and centrifuged at 10,000 g for 20 min. The visible pellet was comprised of white bacteria. The bacteria pellet was resuspended in 25 ml PBS and the presence of bacteria was confirmed by viewing a small aliquot on a glass slide. The number and viability of bacterial cells were determined using the BD Cell Viability and counting Kit (BD Biosciences). Isolated bacterial cells were stained with thiazole orange and propidium iodide, then acquired by a FACS Canto II (BD Biosciences) flow cytometer in the presence of counting beads. Data were processed using Flowjo software, and the concentration of live bacterial cells per mg of stool was calculated. Isolated FBCs were aliquoted and stored at −80°C before use in *in vitro* assays.

### Isolation Method of Individual Bacteria from MSM and HIV+ ART+ Fecal Samples

Diverse bacteria were cultured from two different stool samples, one from an HIV-positive MSM and one from an HIV-negative MSM, using previously described methods.^60^ Specifically, a Poisson model was used to calculate a dilution that would yield ~ 30 isolates per 96 wells with most being single cells. Fecal bacteria were serially diluted into MegaMedia, a complex media that was specifically formulated to support the growth of diverse intestinal bacteria for the development of personalized culture collections.^60^ Wells with observed growth were subjected to barcoded 16S rRNA targeted sequencing as described above for biopsy samples, but using extracted DNA from bacteria in each well as template. All isolates were cryopreserved in 25% glycerol. Sequences were taxonomically assigned using the GreenGenes database 18_3,^61^ and also by BLAST against the cultured component of the Ribosomal Database project to determine relationship to the type strain of *H. biformis*.

### Genomic sequencing and comparisons of *Holdemanella* isolates

The genomes were sequence at the Microbial Genome Sequencing Center (MiGS) (Pittsburgh, PA), on the NextSeq 550 platform following library preparation with the Illumina Nextera kit. Sequences from the type strain of *H. biformis* were downloaded from NCBI (DSM3989). Genomes were then processed using YMP (https://github.com/epruesse/ymp), a customizable metagenomics pipeline developed in the Lozupone lab by Dr. Elmar Pruesse. Through YMP’s interface, we used BBMap to trim reads with a quality score less than 20, and then used UUnicycler^62^ to assemble the reads into contigs. We submitted the draft assemblies to MG-RAST^63^ to get functional annotations. Genome completeness and contamination were estimated using CheckM.^64^ We used PanACoTA (https://github.com/gem-pasteur/PanACoTA)^65^ to define the core and pangenomes. In the core genome step, genes were predicted using Prodigal and the universally persistent features were translated and concatenated. Concatenated protein sequences were aligned using MAFFT^66,67^ and the resulting alignment was used as the basis for FastTree’s phylogeny estimation.

### Bacterial preparation

Bacterial lysates for the type strain of *H. biformis* (ATCC 27806) and *B. uniformis* (ATCC 8492) were purchased from the ATCC. Two more *Holdemanella* strains were isolated from stools from our subjects as described above. These were incubated anaerobically in Balch-type tubes with butyl rubber stoppers at 37°C until stationary phase. When robust culture growth was observed (typically after 18–36 hours depending on the bacteria), liquid cultures were centrifuged at 2500 rpm for 10 minutes. Supernatant was removed and the pellet was resuspended in PBS and frozen at −80°C. Resuspended bacteria were subjected to freeze/thaw and heat-killed for 1 hour at 75°C before being used in immune stimulations. The lysates were then quantified using a BCA protein assay kit (Thermo Scientific), then adjusted to 700 μg/ml and stored at −80°C.

### Immune assays with FBC and bacteria

To quantify CCR5 expression after 24 hours stimulations with FBCs or individual bacterial species, LPMCs were thawed in complete RPMI containing 10 μg/ml DNase I (Sigma-Aldrich), then washed again with complete RPMI before plating. 1 × 10^6^/ml LPMCs were plated per well in a 48-well flat bottom plate. FBC were added to specified wells at 5:1 FBC:LPMC ratio. Single bacterial species were added at 0.1 ug/mL to the wells. Cells were incubated at 37°C, 5% CO_2_ and 95% humidity for 24 hours to allow for stimulation, and then washed twice with complete RPMI.

LPMCs were then stained for 30 min at 4°C by antibodies: FITC labeled anti-CD3 (BioLegend Cat# 317306, clone OKT3), Brilliant Violet 421 labeled anti-CD8 (BioLegend Cat# 344748, clone SK1), PerCP-Cy5.5 labeled anti-CD4 (BioLegend Cat# 317428, clone OKT4), Brilliant Violet 605 labeled anti-CCR5 (BD Biosciences Cat# 742912, clone 2D7) and LIVE/DEAD™ Fixable Aqua Dead Cell Stain Kit (Invitrogen Cat# L34957). Cells were washed and treated with RPMI containing 10 μg/mL DNase I for 10 min at room temperature, then fixed in 1% paraformaldehyde and acquired using a Celesta flow cytometer (BD Biosciences). Unstimulated cells were used as a negative control.

For cytokine secretion quantification, 1 × 10^6^ cells/ml LPMCs were cultured in 48 well plates with 10 μg/ml of bacterial lysates or medium alone. Cultures were incubated at 37°C in a humidified 5% CO_2_ atmosphere for 24 hours and culture supernatants were harvested and centrifuged to remove remaining cells and stored at −80°C. Then, TNF-α and IL-10 levels were assayed using Human ELISA Deluxe Set Human TNF-α and Human ELISA Deluxe Set Human IL-10 (BioLegend). Assays were performed per manufacturer’s instructions and analyzed using a Sector Imager 2400 (Meso Scale Discovery).

### TNF-a/Il-10 titration and CCR5 expression in LPMC

LPMCs (1 × 10^6^ cells/ml) from 3 subjects were stimulated with various combinations of TNF-α (Sigma-Aldrich) and IL-10 (Sigma-Aldrich) using a total concentration of 10 ng/mL (i.e. a 9 ng/mL TNF-α and 1 ng/mL IL-10 combination sample was used as 9:1 TNF-α:IL-10 samples). Unstimulated cells were used as a negative control. The incubation and staining method were the same with above.

### PCR and RT-PCR of *Holdemanella* genes

Primers were selected for the 8 gene targets using the PrimerQuest tool from IDT (Coralville, Iowa, USA) and selected based on target size and melting temperatures. Bacterial cultures were grown for 18 hours, approximately peak log phase. For extraction of DNA, 3 mL of culture was centrifuged at 3000 rpm for 5 minutes at 4°C to pellet cells and then resuspended in 250 µL of EB buffer (Qiagen). All 250 µL of the suspension was extracted using the DNeasy PowerSoil Kit (Qiagen) following the standard extraction protocol with the addition of a 10 min incubation at 65°C following the addition of the C1 reagent. For extraction of RNA, 1.5 mL of culture was centrifuged at 3000 rpm for 5 minutes at 4°C to pellet cells. The pellet was extracted with the PureLink RNA Mini kit (Invitrogen) and cDNA was generated using the RT-PCR Quantitect Reverse Transcription Kit (Qiagen) and 125ng of RNA. The gDNA was normalized to 1ng/µL, the cDNA was used neat and 5 µL of template was used per PCR. The PCR was conducted using a 3 minute hot-start at 94°C and an annealing temperature of 55°C for 30 cycles.

### Statistical analysis for difference in immune cell populations/cytokines

Statistical analysis for difference in immune cell populations and cytokines was performed using GraphPad Prism (GraphPad) for t-test, Kruskal-Wallis test, one-way and two-way ANOVA, and Spearman correlation analysis to determine significance of differences between subject groups. A *p* value of <0.05 was considered statistically significant.

## Supplementary Material

Supplemental Material

## Data Availability

16S rRNA data has been deposited at the European Nuceotide Archive at accession PRJEB48202. Genome sequences have been deposited to NCBI at SAMN22575053 (*H. porci*), and SAMN22575054 (*H. biformis* strain 2).
