# Supplementary material for: Intestinal microbial communities and Holdemanella isolated from HIV+/− men who have sex with men increase frequencies of lamina propria CCR5+ CD4+ T cells
Source: Gut Microbes. 2021 Nov 24;13(1):1997292. doi: 10.1080/19490976.2021.1997292 (PMC8632320; doi:10.1080/19490976.2021.1997292)
Supplement: Supplemental Material [file KGMI_A_1997292_SM5253.docx]

**Supplemental Figure and Figure legend**


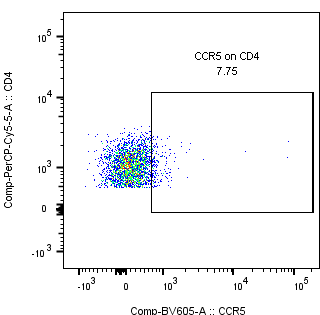

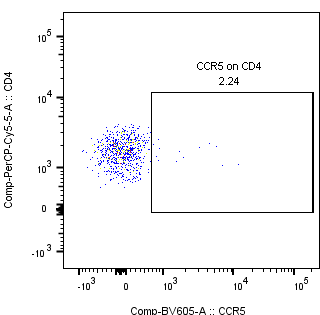

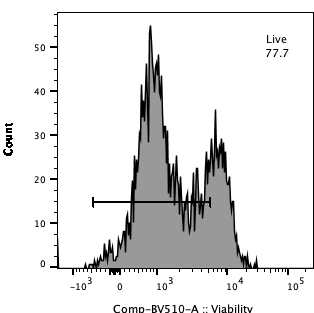

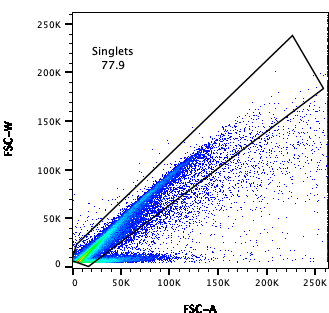

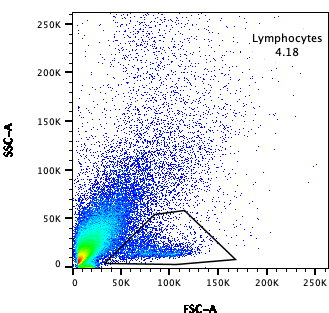

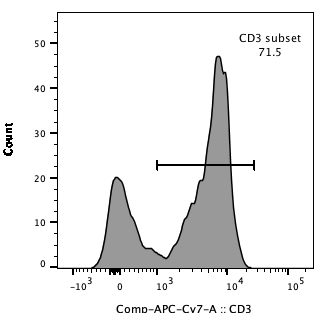

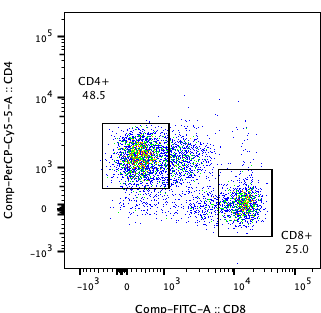


CCR5+ CD4+

7.75%

FSC-A

FSC-W

FSC-A

FSC-A

count

Viability

count

Singlets

Lymphocytes

Live

CD3+

CD3

CD4

CD8

CD4+

CD8+

CCR5

CD4

CD4

HIV- MSW

HIV- MSM

HIV+ ART+

HIV+ ART-

CCR5

Isotype

2.24%


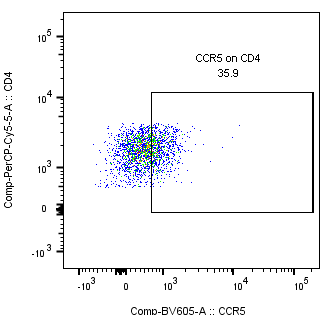

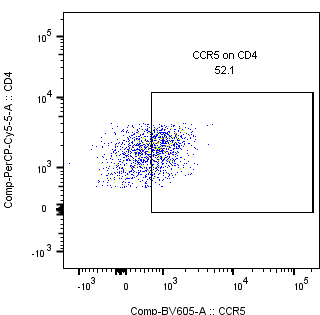

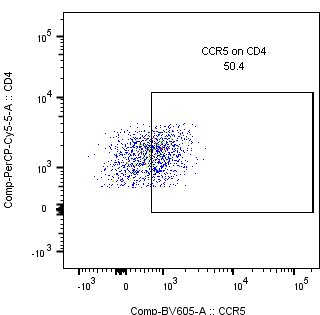


CCR5+ CD4+

35.9%

CCR5

CD4

CCR5+ CD4+

50.4%

CCR5

CD4

CCR5+ CD4+

52.1%

CCR5

CD4

***Supplemental Figure 1. Gating strategy for CD4^+^ CCR5^+^ T cells.***

Representative plots from LPMC stimulated with fecal bacterial communities isolated from HIV- non-MSM and HIV+/− Men of who have Sex with Men (MSM) on and off Antiretroviral therapy (ART) are shown.

| Analysis | Subject group | Sample size | Age median (range) | Race and ethnicity | CD4 count median (cells/μL, range) | Viral load median (copies/mL, range) |
| --- | --- | --- | --- | --- | --- | --- |
| LPMC stimulated with FBC  (Fig. 1) | HIV− MSW | 9 (M5 F4) | 29 (21-41) | 8/0/0/1 | N/A | N/A |
|  | HIV− MSM | 7 (M7 F0) | 47 (29-63)* | 6/1/0/0 | N/A | N/A |
|  | HIV+ ART+ | 7 (M7 F0) | 52 (30-54)** | 5/0/2/0 | 988 (466-1361) | 0 (0-35) |
|  | HIV+ ART− | 9 (M9 F0) | 33 (21-50) | 7/1/1/0 | 497 (403-939) | 146427  (23600-522935) |
| Biopsy  Sequence/  CyTOF  (Fig. 2) | HIV− MSM | 9 (M9 F0) | 41 (26-58) | 6/2/0/1 | N/A | N/A |
|  | HIV+ ART+ | 21 (M21 F0) | 51 (30-65)* | 19/0/2/0 | 753 (190-1437) | 0 (0-40) |

***Supplemental Table 1. Cohort description***

Descriptions of the cohorts used for 1) LPMC stimulations show in in Figure 1 (row 1) and 2) Correlations of microbial taxa with CCR5^+^ CD4^+^ T cells in intestinal biopsy show in Figure 2 (row 2). For race and ethnicity, values are numbers of white/white-hispanic/black/Asian or other study subjects included in each analysis. Significant differences in age in the cohort used for LPMC stimulations are in comparison to the HIV− MSW cohort. Significance tests used the Kruskal-Wallis with multiple comparisons (row 1) and Mann-Whitney (row 2), respectively. * = *p* < 0.05, ** = *p* < 0.01.

| Presence | Category | Subcategory | Subsystem | Role |
| --- | --- | --- | --- | --- |
| Both | Carbohydrates | Monosaccharides | Mannose Metabolism | Mannose-6-phosphate isomerase (EC 5.3.1.8) |
| Both | Carbohydrates | Monosaccharides | Mannose Metabolism | PTS system, mannose-specific IIB component (EC 2.7.1.69) |
| Both | Carbohydrates | Monosaccharides | Mannose Metabolism | Phosphomannomutase (EC 5.4.2.8) |
| Both | Carbohydrates | Monosaccharides | Mannose Metabolism | Putative regulator of the mannose operon, ManO |
| H. biformis Strain 2 | Carbohydrates | Monosaccharides | Mannose Metabolism | PTS system, mannose-specific IIA component (EC 2.7.1.69) |
| Both | Cell Wall and Capsule | Capsular and extracellular polysacchrides | Exopolysaccharide Biosynthesis | Manganese-dependent protein-tyrosine phosphatase (EC 3.1.3.48) |
| Both | Cell Wall and Capsule | Capsular and extracellular polysacchrides | Exopolysaccharide Biosynthesis | Tyrosine-protein kinase EpsD (EC 2.7.10.2) |
| Both | Cell Wall and Capsule | Capsular and extracellular polysacchrides | Exopolysaccharide Biosynthesis | Tyrosine-protein kinase transmembrane modulator EpsC |
| Both | Cell Wall and Capsule | Capsular and extracellular polysacchrides | Polysaccharide deacetylases | Peptidoglycan N-acetylglucosamine deacetylase (EC 3.5.1.-) |
| Both | Cell Wall and Capsule | Capsular and extracellular polysacchrides | Rhamnose containing glycans | Alpha-L-Rha alpha-1,3-L-rhamnosyltransferase (EC 2.4.1.-) |
| Both | Cell Wall and Capsule | Capsular and extracellular polysacchrides | Rhamnose containing glycans | Glucose-1-phosphate thymidylyltransferase (EC 2.7.7.24) |
| Both | Cell Wall and Capsule | Capsular and extracellular polysacchrides | Rhamnose containing glycans | Glycerol-3-phosphate cytidylyltransferase (EC 2.7.7.39) |
| Both | Cell Wall and Capsule | Capsular and extracellular polysacchrides | Rhamnose containing glycans | Teichoic acid export ATP-binding protein TagH (EC 3.6.3.40) |
| Both | Cell Wall and Capsule | Capsular and extracellular polysacchrides | Rhamnose containing glycans | UDP-glucose 4-epimerase (EC 5.1.3.2) |
| Both | Cell Wall and Capsule | Capsular and extracellular polysacchrides | Rhamnose containing glycans | dTDP-4-dehydrorhamnose 3,5-epimerase (EC 5.1.3.13) |
| Both | Cell Wall and Capsule | Capsular and extracellular polysacchrides | Rhamnose containing glycans | dTDP-4-dehydrorhamnose reductase (EC 1.1.1.133) |
| Both | Cell Wall and Capsule | Capsular and extracellular polysacchrides | Rhamnose containing glycans | dTDP-glucose 4,6-dehydratase (EC 4.2.1.46) |
| Both | Cell Wall and Capsule | Capsular and extracellular polysacchrides | dTDP-rhamnose synthesis | Glucose-1-phosphate thymidylyltransferase (EC 2.7.7.24) |
| Both | Cell Wall and Capsule | Capsular and extracellular polysacchrides | dTDP-rhamnose synthesis | dTDP-4-dehydrorhamnose 3,5-epimerase (EC 5.1.3.13) |
| Both | Cell Wall and Capsule | Capsular and extracellular polysacchrides | dTDP-rhamnose synthesis | dTDP-4-dehydrorhamnose reductase (EC 1.1.1.133) |
| Both | Cell Wall and Capsule | Capsular and extracellular polysacchrides | dTDP-rhamnose synthesis | dTDP-glucose 4,6-dehydratase (EC 4.2.1.46) |
| H. porci | Cell Wall and Capsule | Capsular and extracellular polysacchrides | Exopolysaccharide Biosynthesis | Exopolysaccharide biosynthesis transcriptional activator EpsA |
| H. porci | Cell Wall and Capsule | Capsular and extracellular polysacchrides | Rhamnose containing glycans | Heteropolysaccharide repeat unit export protein |
| H. biformis Strain 2 | Cell Wall and Capsule | Capsular and extracellular polysacchrides | Exopolysaccharide Biosynthesis | Glycosyl transferase, group 2 family protein |
| H. biformis Strain 2 | Cell Wall and Capsule | Capsular and extracellular polysacchrides | Exopolysaccharide Biosynthesis | Undecaprenyl-phosphate galactosephosphotransferase (EC 2.7.8.6) |
| H. biformis Strain 2 | Cell Wall and Capsule | Capsular and extracellular polysacchrides | dTDP-rhamnose synthesis | dTDP-rhamnosyl transferase RfbF (EC 2.-.-.-) |
| Both | Cell Wall and Capsule | Gram-Positive cell wall components | Sortase | NPQTN specific sortase B |
| Both | Cell Wall and Capsule | Gram-Positive cell wall components | Sortase | Sortase A, LPXTG specific |
| Both | Cell Wall and Capsule | Gram-Positive cell wall components | Teichoic and lipoteichoic acids biosynthesis | 2-C-methyl-D-erythritol 4-phosphate cytidylyltransferase (EC 2.7.7.60) |
| Both | Cell Wall and Capsule | Gram-Positive cell wall components | Teichoic and lipoteichoic acids biosynthesis | CDP-glycerol: N-acetyl-beta-D-mannosaminyl-1,4-N-acetyl-D-glucosaminyldiphosphoundecaprenyl glycerophosphotransferase |
| Both | Cell Wall and Capsule | Gram-Positive cell wall components | Teichoic and lipoteichoic acids biosynthesis | CDP-glycerol:poly(glycerophosphate) glycerophosphotransferase (EC 2.7.8.12) |
| Both | Cell Wall and Capsule | Gram-Positive cell wall components | Teichoic and lipoteichoic acids biosynthesis | Regulation of D-alanyl-lipoteichoic acid biosynthesis, DltR |
| Both | Cell Wall and Capsule | Gram-Positive cell wall components | Teichoic and lipoteichoic acids biosynthesis | Teichoic acid export ATP-binding protein TagH (EC 3.6.3.40) |
| Both | Cell Wall and Capsule | Gram-Positive cell wall components | Teichoic and lipoteichoic acids biosynthesis | Teichoic acid glycosylation protein |
| Both | Cell Wall and Capsule | Gram-Positive cell wall components | Teichoic and lipoteichoic acids biosynthesis | Teichoic acid translocation permease protein TagG |
| H. porci | Cell Wall and Capsule | Gram-Positive cell wall components | Sortase | Cell wall surface anchor family protein |
| H. biformis Strain 2 | Cell Wall and Capsule | Gram-Positive cell wall components | Polyglycerolphosphate lipoteichoic acid biosynthesis | Glycosyltransferase LafA, responsible for the formation of Glc-DAG |
| H. biformis Strain 2 | Cell Wall and Capsule | Gram-Positive cell wall components | Teichoic and lipoteichoic acids biosynthesis | N-acetylmannosaminyltransferase (EC 2.4.1.187) |
| H. biformis Strain 2 | Cell Wall and Capsule | Gram-Positive cell wall components | Teichuronic acid biosynthesis | Putative N-acetylgalactosaminyl-diphosphoundecaprenol glucuronosyltransferase |
| Both | Carbohydrates | Monosaccharides | Mannose Metabolism | Mannose-6-phosphate isomerase (EC 5.3.1.8) |
| Both | Carbohydrates | Monosaccharides | Mannose Metabolism | PTS system, mannose-specific IIB component (EC 2.7.1.69) |
| Both | Carbohydrates | Monosaccharides | Mannose Metabolism | Phosphomannomutase (EC 5.4.2.8) |
| Both | Carbohydrates | Monosaccharides | Mannose Metabolism | Putative regulator of the mannose operon, ManO |
| H. biformis Strain 2 | Carbohydrates | Monosaccharides | Mannose Metabolism | PTS system, mannose-specific IIA component (EC 2.7.1.69) |
| Both | Cell Wall and Capsule | Capsular and extracellular polysacchrides | Exopolysaccharide Biosynthesis | Manganese-dependent protein-tyrosine phosphatase (EC 3.1.3.48) |
| Both | Cell Wall and Capsule | Capsular and extracellular polysacchrides | Exopolysaccharide Biosynthesis | Tyrosine-protein kinase EpsD (EC 2.7.10.2) |
| Both | Cell Wall and Capsule | Capsular and extracellular polysacchrides | Exopolysaccharide Biosynthesis | Tyrosine-protein kinase transmembrane modulator EpsC |
| Both | Cell Wall and Capsule | Capsular and extracellular polysacchrides | Polysaccharide deacetylases | Peptidoglycan N-acetylglucosamine deacetylase (EC 3.5.1.-) |
| Both | Cell Wall and Capsule | Capsular and extracellular polysacchrides | Rhamnose containing glycans | Alpha-L-Rha alpha-1,3-L-rhamnosyltransferase (EC 2.4.1.-) |
| Both | Cell Wall and Capsule | Capsular and extracellular polysacchrides | Rhamnose containing glycans | Glucose-1-phosphate thymidylyltransferase (EC 2.7.7.24) |
| Both | Cell Wall and Capsule | Capsular and extracellular polysacchrides | Rhamnose containing glycans | Glycerol-3-phosphate cytidylyltransferase (EC 2.7.7.39) |
| Both | Cell Wall and Capsule | Capsular and extracellular polysacchrides | Rhamnose containing glycans | Teichoic acid export ATP-binding protein TagH (EC 3.6.3.40) |
| Both | Cell Wall and Capsule | Capsular and extracellular polysacchrides | Rhamnose containing glycans | UDP-glucose 4-epimerase (EC 5.1.3.2) |
| Both | Cell Wall and Capsule | Capsular and extracellular polysacchrides | Rhamnose containing glycans | dTDP-4-dehydrorhamnose 3,5-epimerase (EC 5.1.3.13) |
| Both | Cell Wall and Capsule | Capsular and extracellular polysacchrides | Rhamnose containing glycans | dTDP-4-dehydrorhamnose reductase (EC 1.1.1.133) |
| Both | Cell Wall and Capsule | Capsular and extracellular polysacchrides | Rhamnose containing glycans | dTDP-glucose 4,6-dehydratase (EC 4.2.1.46) |
| Both | Cell Wall and Capsule | Capsular and extracellular polysacchrides | dTDP-rhamnose synthesis | Glucose-1-phosphate thymidylyltransferase (EC 2.7.7.24) |
| Both | Cell Wall and Capsule | Capsular and extracellular polysacchrides | dTDP-rhamnose synthesis | dTDP-4-dehydrorhamnose 3,5-epimerase (EC 5.1.3.13) |
| Both | Cell Wall and Capsule | Capsular and extracellular polysacchrides | dTDP-rhamnose synthesis | dTDP-4-dehydrorhamnose reductase (EC 1.1.1.133) |
| Both | Cell Wall and Capsule | Capsular and extracellular polysacchrides | dTDP-rhamnose synthesis | dTDP-glucose 4,6-dehydratase (EC 4.2.1.46) |
| H. porci | Cell Wall and Capsule | Capsular and extracellular polysacchrides | Exopolysaccharide Biosynthesis | Exopolysaccharide biosynthesis transcriptional activator EpsA |
| H. porci | Cell Wall and Capsule | Capsular and extracellular polysacchrides | Rhamnose containing glycans | Heteropolysaccharide repeat unit export protein |
| H. biformis Strain 2 | Cell Wall and Capsule | Capsular and extracellular polysacchrides | Exopolysaccharide Biosynthesis | Glycosyl transferase, group 2 family protein |
| H. biformis Strain 2 | Cell Wall and Capsule | Capsular and extracellular polysacchrides | Exopolysaccharide Biosynthesis | Undecaprenyl-phosphate galactosephosphotransferase (EC 2.7.8.6) |
| H. biformis Strain 2 | Cell Wall and Capsule | Capsular and extracellular polysacchrides | dTDP-rhamnose synthesis | dTDP-rhamnosyl transferase RfbF (EC 2.-.-.-) |
| Both | Cell Wall and Capsule | Gram-Positive cell wall components | Sortase | NPQTN specific sortase B |
| Both | Cell Wall and Capsule | Gram-Positive cell wall components | Sortase | Sortase A, LPXTG specific |
| Both | Cell Wall and Capsule | Gram-Positive cell wall components | Teichoic and lipoteichoic acids biosynthesis | 2-C-methyl-D-erythritol 4-phosphate cytidylyltransferase (EC 2.7.7.60) |
| Both | Cell Wall and Capsule | Gram-Positive cell wall components | Teichoic and lipoteichoic acids biosynthesis | CDP-glycerol: N-acetyl-beta-D-mannosaminyl-1,4-N-acetyl-D-glucosaminyldiphosphoundecaprenyl glycerophosphotransferase |
| Both | Cell Wall and Capsule | Gram-Positive cell wall components | Teichoic and lipoteichoic acids biosynthesis | CDP-glycerol:poly(glycerophosphate) glycerophosphotransferase (EC 2.7.8.12) |
| Both | Cell Wall and Capsule | Gram-Positive cell wall components | Teichoic and lipoteichoic acids biosynthesis | Regulation of D-alanyl-lipoteichoic acid biosynthesis, DltR |
| Both | Cell Wall and Capsule | Gram-Positive cell wall components | Teichoic and lipoteichoic acids biosynthesis | Teichoic acid export ATP-binding protein TagH (EC 3.6.3.40) |
| Both | Cell Wall and Capsule | Gram-Positive cell wall components | Teichoic and lipoteichoic acids biosynthesis | Teichoic acid glycosylation protein |
| Both | Cell Wall and Capsule | Gram-Positive cell wall components | Teichoic and lipoteichoic acids biosynthesis | Teichoic acid translocation permease protein TagG |
| H. porci | Cell Wall and Capsule | Gram-Positive cell wall components | Sortase | Cell wall surface anchor family protein |
| H. biformis Strain 2 | Cell Wall and Capsule | Gram-Positive cell wall components | Polyglycerolphosphate lipoteichoic acid biosynthesis | Glycosyltransferase LafA, responsible for the formation of Glc-DAG |
| H. biformis Strain 2 | Cell Wall and Capsule | Gram-Positive cell wall components | Teichoic and lipoteichoic acids biosynthesis | N-acetylmannosaminyltransferase (EC 2.4.1.187) |
| H. biformis Strain 2 | Cell Wall and Capsule | Gram-Positive cell wall components | Teichuronic acid biosynthesis | Putative N-acetylgalactosaminyl-diphosphoundecaprenol glucuronosyltransferase |

***Supplemental Table 2. Actual genes description***

Actual genes that differ and relationships across *H. porci* and *H. biformis* strain 2.
